# Eating two larger meals a day (breakfast and lunch) is more effective than six smaller meals in a reduced-energy regimen for patients with type 2 diabetes: a randomised crossover study

**DOI:** 10.1007/s00125-014-3253-5

**Published:** 2014-05-18

**Authors:** Hana Kahleova, Lenka Belinova, Hana Malinska, Olena Oliyarnyk, Jaroslava Trnovska, Vojtech Skop, Ludmila Kazdova, Monika Dezortova, Milan Hajek, Andrea Tura, Martin Hill, Terezie Pelikanova

**Affiliations:** 1 Diabetes Centre, Institute for Clinical and Experimental Medicine, Videnska 1958/9, 140 21 Prague, Czech Republic; 2 First Faculty of Medicine, Charles University, Prague, Czech Republic; 3 Department of Diagnostic and Interventional Radiology, Institute for Clinical and Experimental Medicine, Prague, Czech Republic; 4 Metabolic Unit, Institute of Biomedical Engineering, National Research Council, Padua, Italy; 5 Department of Steroid Hormones and Proteohormones, Institute of Endocrinology, Prague, Czech Republic

**Keywords:** Hepatic fat content, Insulin sensitivity, Two meals a day, Type 2 diabetes

## Abstract

**Aims/hypothesis:**

The aim of the study was to compare the effect of six (A6 regimen) vs two meals a day, breakfast and lunch (B2 regimen), on body weight, hepatic fat content (HFC), insulin resistance and beta cell function.

**Methods:**

In a randomised, open, crossover, single-centre study (conducted in Prague, Czech Republic), we assigned 54 patients with type 2 diabetes treated with oral hypoglycaemic agents, both men and women, age 30–70 years, BMI 27–50 kg/m^2^ and HbA_1c_ 6–11.8% (42–105 mmol/mol), to follow two regimens of a hypoenergetic diet, A6 and B2, each for 12 weeks. Randomisation and allocation to trial groups (*n* = 27 and *n* = 27) were carried out by a central computer system. Individual calculations of energy requirements for both regimens were based on the formula: (resting energy expenditure × 1.5) − 2,092 kJ. The diet in both regimens had the same macronutrient and energy content. HFC was measured by proton magnetic resonance spectroscopy. Insulin sensitivity was measured by isoglycaemic–hyperinsulinaemic clamp and calculated by mathematical modelling as oral glucose insulin sensitivity (OGIS). Beta cell function was assessed during standard meal tests by C-peptide deconvolution and was quantified with a mathematical model. For statistical analysis, 2 × 2 crossover ANOVA was used.

**Results:**

The intention-to-treat analysis included all participants (*n* = 54). Body weight decreased in both regimens (*p* < 0.001), more for B2 (−2.3 kg; 95% CI −2.7, −2.0 kg for A6 vs −3.7 kg; 95% CI −4.1, −3.4 kg for B2; *p* < 0.001). HFC decreased in response to both regimens (*p* < 0.001), more for B2 (−0.03%; 95% CI −0.033%, −0.027% for A6 vs −0.04%; 95% CI −0.041%, −0.035% for B2; *p* = 0.009). Fasting plasma glucose and C-peptide levels decreased in both regimens (*p* < 0.001), more for B2 (*p* = 0.004 and *p* = 0.04, respectively). Fasting plasma glucagon decreased with the B2 regimen (*p* < 0.001), whereas it increased (*p* = 0.04) for the A6 regimen (*p* < 0.001). OGIS increased in both regimens (*p* < 0.01), more for B2 (*p* = 0.01). No adverse events were observed for either regimen.

**Conclusions/interpretation:**

Eating only breakfast and lunch reduced body weight, HFC, fasting plasma glucose, C-peptide and glucagon, and increased OGIS, more than the same caloric restriction split into six meals. These results suggest that, for type 2 diabetic patients on a hypoenergetic diet, eating larger breakfasts and lunches may be more beneficial than six smaller meals during the day.

*Trial registration* ClinicalTrials.gov number, NCT01277471, completed.

*Funding* Grant NT/11238-4 from Ministry of Health, Prague, Czech Republic and the Agency of Charles University – GAUK No 702312.

## Introduction

Frequency of meals is an important aspect of nutrition, with profound effects on human health and lifespan. Excessive energy intake is associated with an increased incidence of chronic diseases including diabetes and is a leading cause of disability and death in Western countries [1]. A hypoenergetic diet is crucial for both the prevention and treatment of type 2 diabetes. It is usually consumed as five or six small meals per day. Eating more frequently is presumed to reduce hunger and thus reduce energy intake and body weight. However, the effects of meal frequency on human health and longevity are unclear [2].

Reduced meal frequency can prevent the development of chronic diseases and extend the lifespan in laboratory animals due to lower oxidative damage and higher stress resistance [3, 4]. Mice under time-restricted feeding have an equivalent energy intake from a high-fat diet as those with ad libitum access yet are protected against obesity, hyperinsulinaemia and hepatic steatosis [5, 6]. Intermittent fasting leads to a prolonged lifespan and positively affects glucose tolerance, insulin sensitivity and incidence of type 2 diabetes in mice [3, 4]. There is also emerging literature demonstrating a relationship between the timing of feeding and weight regulation in animals.

Observational trials in humans indicate that eating more often than three times a day may play a role in overweight and obesity [7] and that frequent eating predisposes to a higher energy intake by increasing food stimuli and difficulty controlling energy balance [8]. In a randomised controlled study, more frequent eating was not related to a greater reduction in energy intake or body weight [9]. In type 2 diabetic patients it has been demonstrated that it may be more beneficial for glycaemic control to eat one larger instead of two smaller meals, provided the diet is rich in fibre [10].

It has been demonstrated that a large isocaloric mixed meal causes a greater postprandial thermogenic response than the same food consumed in six smaller portions [11]. Observational data suggest that eating meals later in the day may influence the success of weight-loss therapy, even in humans [12]. It has also been shown that fat storage increases during the day and is the greatest after an evening meal [13]. It has been observed that eating breakfast regularly may protect against weight gain, despite a higher total daily energy intake [14].

To the best of our knowledge, no interventional trials have investigated the relationship between eating frequency and weight change together with hepatic fat content (HFC), glucose tolerance and insulin resistance in humans, especially in patients with type 2 diabetes. The aim of our study was to compare the effect of six vs two meals a day (breakfast and lunch, as this regimen allows a reasonable fasting time, yet is sustainable in the long term) with the same caloric restriction on body weight, HFC, insulin resistance and beta cell function in individuals with type 2 diabetes. It was hypothesised that eating only breakfast and lunch would reduce body weight and HFC (and consequently, improve insulin resistance and beta cell function) more than six meals a day would.

## Methods

### Participants

Out of the 219 individuals screened, 54 patients with type 2 diabetes (with disease duration of more than 1 year) treated by oral hypoglycaemic agents (both men and women), age 30–70 years, BMI 27–50 kg/m^2^ and HbA_1c_ 6–11.8% (42–105 mmol/mol), met all the inclusion criteria, gave their written informed consent and underwent randomisation. Exclusion criteria comprised alcohol or drug abuse, pregnancy or lactation, unstable medication or weight in the last 3 months, a diagnosis of type 1 diabetes and the presence of a cardiostimulant.

### Study design

**Fig. 1:**
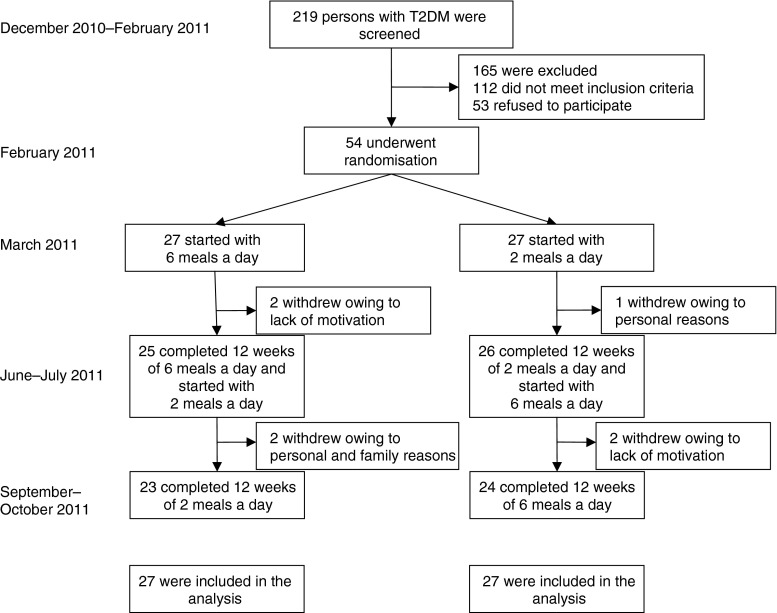
Enrolment of the participants and completion of the study. T2DM, type 2 diabetes mellitus

We used a randomised crossover study design. The study protocol was approved by the Institutional Ethical Committee. In a single-centre study, after a 1 month run-in period (when the patients learned how to write their food diaries and use the pedometers and glucometers), the participants began a 12 week regimen of either six (A6) or two (B2) meals a day. The A6 regimen consisted of three main meals (breakfast, lunch and dinner), and three smaller snacks in between. The B2 regimen consisted of breakfast (eaten between 06:00 and 10:00 hours) and lunch (eaten between 12:00 and 16:00 hours). The regimens were switched for the subsequent 12 weeks. All measurements were performed at weeks 0 (baseline), 12 and 24 (Fig. 1 and Table 1).

**Table 1:** Baseline characteristics of the study population

Characteristic	Study group (*n* = 54)
Age (years)	59.4 ± 7.0
Sex, *n* (%)	
Male	29 (54)
Female	25 (46)
Duration of diabetes (years)	8.1 ± 5.8
Smokers, *n* (%)	10 (19)
Weight (kg)	94.1 ± 15.5
BMI (kg/m^−2^)	32.6 ± 4.9
HbA_1c_ (DCCT) (%)	7.2 ± 3.3
HbA_1c_ (IFCC) (mmol/mol)	54.9 ± 13.0
Systolic blood pressure (mmHg)	140 ± 14
Diastolic blood pressure (mmHg)	85 ± 8
Resting heart rate (beats/min)	71 ± 9
Oral hypoglycaemic agents, *n* (%)	
Metformin	41 (76)
Sulfonylurea	16 (30)
Thiazolidinedione	3 (6)
Glinides	2 (4)
Acarbose	1 (2)
DPP-4 inhibitors	19 (35)
Lipid-lowering therapy, *n* (%)	31 (57)
Antihypertensive therapy, *n* (%)	33 (61)

Data are means ± SD

DCCT, Diabetes Control and Complications Trial; IFCC, International Federation of Clinical Chemistry

### Diet

The composition of the diet in both regimens followed the Study Group on Diabetes and Nutrition of the European Association for the Study of Diabetes guidelines [15] with the same caloric restriction: a restriction of 2,092 kJ/day (500 kcal/day) based on the measurement of each individual’s resting energy expenditure (REE) by indirect calorimetry (metabolic monitor VMAX; SensorMedics, Anaheim, CA, USA) [16]. Individual calculations of energy requirements for both regimens were based on the formula: (REE × 1.5) − 2,092 kJ. The diet derived 50–55% of its total energy from carbohydrates, 20–25% from protein and less than 30% from fat (≤7% saturated fat, less than 200 mg/day of cholesterol), with 30–40 g/day of fibre. Alcoholic beverages were limited to one per day for women and two per day for men. Participants were asked not to alter their exercise habits during the study. Each regimen started with a 4 day tutorial where they learned in detail how to compose and prepare their diet, with follow-up 1 h weekly meetings with lectures and cooking classes throughout the whole study. All the meals during the entire 24 weeks of the study were provided for one half of the participants (randomised within each study arm with an equal number of participants) while the other half of the participants prepared their meals by themselves.

### Compliance

At weeks 0, 12, and 24, a 3 day dietary record (2 weekdays and 1 weekend day) was completed by each participant. A registered dietitian analysed all these dietary records using a country-specific food-nutrient database NutriDan 1.2 (www.institut-danone.cz/cz/odborna-sekce/nutridan).

### Physical activity

This was assessed with an Omron HJ-720IT pedometer (Omron, Kyoto, Japan; using a 1 month average step count for evaluation) and two questionnaires: the International Physical Activity Questionnaire [17] and the Baecke questionnaire [18] at weeks 0, 12, and 24.

### Medication

Participants were asked to continue their pre-existing medication regimens, except when hypoglycaemia occurred repeatedly (fasting plasma glucose determined at the laboratory <4.4 mmol/l or a capillary glucose reading <3.4 mmol/l accompanied by hypoglycaemic symptoms). In such cases, medications were reduced by a study physician following the medication protocol. All participants were given an Accu-Chek Performa glucometer (Roche, Basel, Switzerland) and instructed how to use it.

### Procedures

All measurements were performed on an outpatient basis at weeks 0, 12 and 24, after a 10–12 h overnight fasting with tap water ad libitum. Height and weight were measured using a periodically calibrated scale accurate to 0.1 kg. Waist circumference was measured with a tape measure placed at the midpoint between the lowest rib and the upper part of the iliac bone. Blood pressure and heart rate were measured after 5 min in a seated position at rest, using a digital M6 Comfort monitor (Omron, Kyoto, Japan). Three measurements were taken 2 min apart. The first measurement was discarded, and the mean of the remaining two measurements was recorded.

#### Indirect calorimetry

Gas exchange measurements were taken during a 45 min basal period before the clamp. Air flow and O_2_ and CO_2_ concentrations in expired and inspired air were measured by a continuous open-circuit system (metabolic monitor VMAX; SensorMedics, Anaheim, CA, USA).

#### Meal tests

Plasma concentrations of glucose, immunoreactive insulin and C-peptide were measured at 0, 30, 60, 120 and 180 min after a standard breakfast (1,895 kJ, 45% carbohydrates, 17% proteins, 38% lipids). Insulin secretion and whole-body insulin sensitivity were calculated by mathematical modelling (described below).

#### Hyperinsulinaemic isoglycaemic clamp

The hyperinsulinaemic (1 mU kg^−1^ min^−1^) isoglycaemic clamp, lasting 3 h, was conducted as previously described [19]. Insulin sensitivity was estimated as the metabolic clearance rate of glucose (MCR) [19].

#### Proton magnetic resonance spectroscopy

HFC was measured by proton magnetic resonance spectroscopy on a 3 T MR scanner (Magnetom Trio, Siemens, Erlangen, Germany) with an eight-channel body array coil. This method has been validated at our institution [20]. The measurement protocol included conventional MRI using a localiser and HASTE sequence with breath-holding in the coronal and transversal planes. Spectra were obtained from three different segments of the right lobe of the liver—volume of interest, 30 ml each and evaluated using the LCModel (www.s-provencher.com/pages/lcmodel.shtml) and MestReC (Mestrelab Research, Santiago de Compostela, Spain) programs. The signal intensities of water and hepatic lipids were used to determine the fat to total signal peak area ratio and then converted to absolute concentrations expressed as a percentage of fat using equations validated by Longo et al [21]. Fourteen individuals did not undergo an HFC measurement due to the patient’s refusal, claustrophobia or the patient’s weight exceeding the limit of the equipment.

### Calculations

Modelling analysis of beta cell function was performed during standard meal tests. Insulin secretory rates were calculated from plasma C-peptide levels by deconvolution [22] and expressed per square meter of estimated body surface area. The dependence of insulin secretory rates on glucose levels was modelled separately for each patient and each study day. The beta cell model used in the present study, describing the relationship between insulin secretion and glucose concentration, has previously been described in detail [23–25].

Briefly, insulin secretion consists of two components. The first component represents the dependence of insulin secretion on absolute glucose concentration at any time point and is characterised by a dose–response function. Characteristic variables of the dose–response are insulin secretion at a fixed glucose concentration and the mean slope in the observed glucose range. The dose–response was modulated by a potentiation factor that accounts for several agents (prolonged exposure to hyperglycaemia, non-glucose substrates, gastrointestinal hormones and neurotransmitters). The potentiation factor was set to be a positive function of time and to be an average of 1 during the experiment. It thus expresses a relative potentiation of the secretory response to glucose.

The second insulin secretion component represents a dynamic dependence of insulin secretion on the rate of change of glucose concentration. Termed the derivative component, it is described by a single variable, rate sensitivity. This secretion component is related to early insulin release [23, 24].

The model variables (the variables of the dose–response, the rate sensitivity and the potentiation factor) were estimated from the glucose and C-peptide concentrations by regularised least squares, as previously described [23, 24]. Estimation of the individual model variables was performed blinded for the randomisation of the patients for treatment.

Whole-body insulin sensitivity was estimated in two ways: (1) as the MCR calculated during the last 20 min of the isoglycaemic hyperinsulinaemic clamp after correction for changes in glucose pool size [19], and (2) by a glucose–insulin model to derive an oral glucose insulin sensitivity (OGIS) index, validated against the clamp data [3].

### Analytical methods

Serum glucose was analysed using the Beckman Analyser glucose-oxidase method (Beckman Instruments, Fullerton, CA, USA). Plasma immunoreactive insulin and C-peptide concentrations were determined using insulin and C-peptide IRMA kits (Immunotech, Prague, Czech Republic). HbA_1c_ was measured by HPLC (Tosoh, Tokyo, Japan). Plasma concentrations of glucagon were measured using ELISA kits (BioVendor, Brno, Czech Republic). Plasma lipids concentrations were measured by enzymatic methods (Roche, Basel, Switzerland). HDL-cholesterol was measured after double precipitation with dextran and MgCl_2_. LDL-cholesterol was estimated using the Friedewald equation if the triacylglycerol concentration was <4.53 mmol/l.

### Statistical analyses

The intention-to-treat analysis included all participants. We tested the distributions of the data. If the distribution was skewed, we used the Box-Cox transformation to attain data symmetry and homoscedasticity [26]. Non-homogeneities in the data were detected using residual analysis as described elsewhere [27]. 2 × 2 crossover ANOVA was used for data evaluation. The model consisted of the between-subject factor ‘sequence’, the factor ‘subject’ and within-subject factors of ‘period’ and ‘treatment’. In a subsequent subanalysis, the factor for prepared meals that were collected by patients was added. The relationships between continuous variables were evaluated using Pearson’s correlation and BMI-adjusted partial correlations.

## Results

**Fig. 2:**
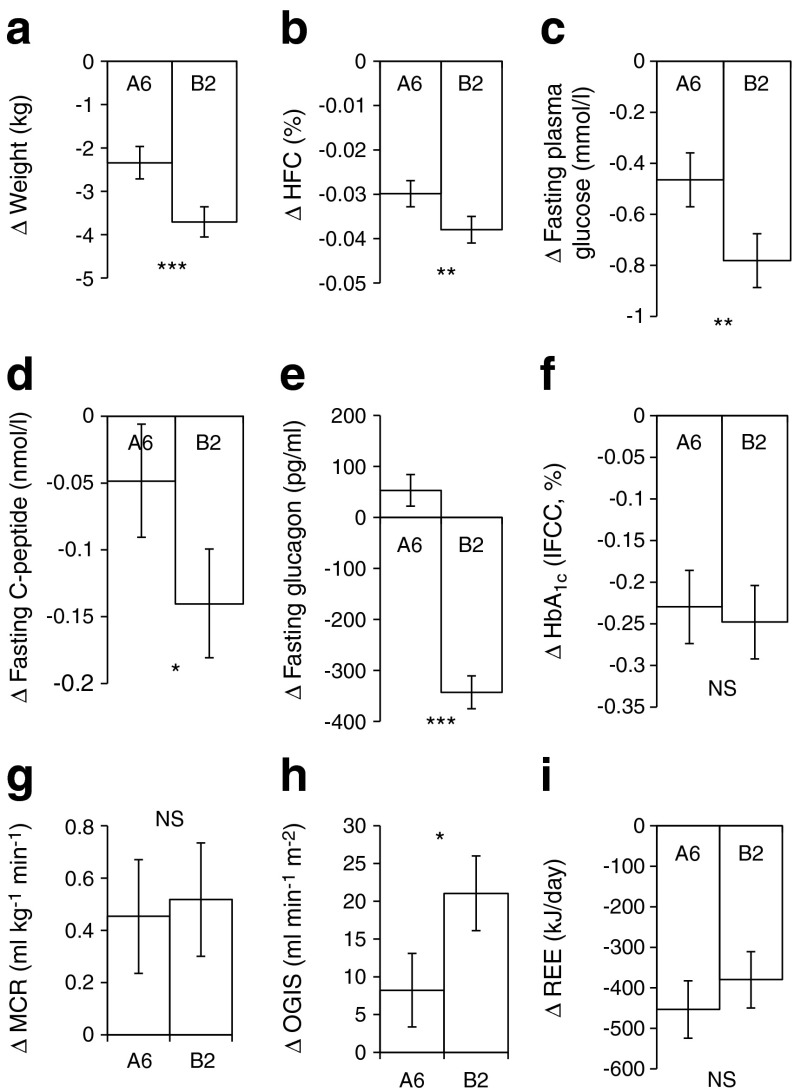
Changes in anthropometric and laboratory variables. Data are shown as changes from baseline in response to the regimen of six (A6) and two meals (B2) a day. Data are mean ± 95% CI. Significance of the factor treatment (assessed by 2 × 2 crossover ANOVA) is indicated by: *p < 0.05; **p < 0.01; ***p < 0.001; NS, non-significant. (**a**) Δ Weight, *n* = 54, (**b**) Δ HFC, *n* = 48, (**c**) Δ Fasting plasma glucose, *n* = 54, (**d**) Δ Fasting plasma C-peptide, *n* = 54, (**e**) Δ Fasting plasma glucagon, *n* = 54, (**f**) Δ HbA_1c_, *n* = 54, (**g**) Δ MCR, *n* = 49, (**h**) Δ Insulin sensitivity (OGIS), *n* = 51, (**i**) Δ REE, *n* = 52. To convert values for HbA_1c_ in % into mmol/mol, subtract 2.15 and multiply by 10.929

The results are expressed as the changes in response to the A6 and B2 regimens, presented as means with 95% CIs (Fig. 2 and Table 2). The factors ‘period’ and ‘sequence’ (the order of the regimens) were not significant. No substantial unfavourable effects of the regimens were observed.

**Table 2:** Changes in anthropometric and laboratory variables in response to regimens of six (A6) and two (B2) meals a day

Variables	Six meals a day (A6)	Two meals a day (B2)	*p* values
Dietary intake – energy (kJ/day)	−1,590 (−1,970 to −1,054)***	−1,757 (−2,105 to −1,201)***	0.731
Dietary intake – fat (g/day)	−35.0 (−43.7 to −27.9)***	−38.2 (−44.4 to −32.2)***	0.921
Dietary intake – carbohydrates (g/day)	−19.7 (−32.4 to −3.1)***	−23.2 (−37.8 to −8.5)***	0.873
Dietary intake – protein (g/day)	+0.9 (−3.7 to +5.6)	−4.6 (−8.7 to +1.1)	0.637
Step count (steps/month)	+2,092 (+879 to +3,493)*	+2,213 (+996 to +3,612)*	0.876
BMI (kg/m^2^)	−0.82 (−0.94 to −0.69)***	−1.23 (−1.4 to −1.17)***	<0.001
Waist circumference (cm)	−1.37 (−2.01 to −0.73)***	−5.14 (−5.78 to −4.50)***	<0.001
Fasting plasma glucose (mmol/l)	−0.47 (−0.57 to −0.36)***	−0.78 (−0.89 to −0.68)***	0.004
Fasting immunoreactive insulin (pmol/l)	−0.69 (−1.18 to −0.21)*	−0.75 (−1.23 to −0.27)*	0.910
Triacylglycerols (mmol/l)	−0.28 (−0.39 to −0.17)**	−0.17 (−0.28 to −0.06)*	0.300
Total cholesterol (mmol/l)	−0.05 (−0.13 to +0.04)	−0.07 (−0.15 to +0.01)	0.730
HDL-cholesterol (mmol/l)	+0.016 (−0.006 to +0.038)	+0.003 (−0.019 to +0.025)	0.570
LDL-cholesterol (mmol/l)	−0.08 (−0.15 to −0.01)*	−0.06 (−0.13 to −0.01)	0.823
Insulin secretion at reference level (pmol min^−1^ m^−2^)	+22.9 (+11.7 to +34.0)*	+20.0 (+8.7 to +31.2)*	0.795
Glucose sensitivity (pmol min^−1^ m^−2^ mmol^−1^ l^−1^)	+5.8 (+2.3 to +9.5)*	+5.9 (+2.3 to +9.5)*	0.991
Rate sensitivity (pmol m^−2^ mmol^−1^ l^−1^)	−141.9 (−248.2 to −36.8)*	−251.5 (−358.9 to −145.3)*	0.303
Potentiation factor (dimensionless)	−0.034 (−0.059 to −0.009)*	−0.038 (−0.062 to −0.013)*	0.890

Data are mean ± 95% CI

Listed *p* values are from 2 × 2 crossover ANOVA for the factor ‘treatment’

Significant changes during the regimens are indicated by: *p < 0.05; **p < 0.01; ***p < 0.001

### Dietary intake and physical activity

Reported dietary intake decreased (*p* < 0.001) comparably under both regimens. Physical activity increased (*p* < 0.05) slightly, but negligibly—by about 2,000 steps per month—in both regimens (see Table 2).

### Body weight and HFC

Body weight and HFC decreased under both regimens (*p* < 0.001), more with B2 (*p* < 0.001; −2.3 kg; 95% CI −2.7, −2.0 kg with A6 vs −3.7 kg; 95% CI −4.1, −3.4 kg with B2; and *p* = 0.009; −0.03%; 95% CI −0.033, −0.027% with A6 vs −0.04%; 95% CI −0.041, −0.035% with B2, respectively; Fig. 2a and b). Similarly, BMI and waist circumference decreased with both regimens (*p* < 0.001), more with B2 (*p* < 0.001; −0.82 kg/m^2^; 95% CI −0.94, −0.69 kg/m^2^ with A6 vs −1.23 kg/m^2^; 95% CI −1.4, −1.17 kg/m^2^ with B2; and −1.37 cm; 95% CI −2.01, −0.73 cm with A6 vs −5.14 cm; 95% CI −5.78, −4.50 cm in B2, respectively; Table 2).

### Glycaemic control

Fasting plasma glucose decreased under both regimens (*p* < 0.001), more with B2 (*p* = 0.004; −0.47 mmol/l; 95% CI −0.57, −0.36 mmol/l with A6 vs −0.78 mmol/l; 95% CI −0.89, −0.68 mmol/l with B2; Fig. 2c). Fasting C-peptide decreased in both regimens, more with B2 (*p* = 0.04; −0.049 nmol/l; 95% CI −0.091, −0.006 nmol/l with A6 vs *p* < 0.001; −0.14 nmol/l 95% CI −0.181, −0.099 nmol/l with B2; *p* = 0.04; Fig. 2d). Fasting immunoreactive insulin decreased (*p* < 0.04) comparably with both regimens (−0.69 pmol/l; 95% CI −1.18, −0.21 pmol/l with A6 vs −0.75 pmol/l; 95% CI −1.23, −0.27 pmol/l with B2; *p* = 0.9; Table 2). Fasting glucagon decreased with B2 (*p* < 0.001; −343 pg/ml; 95% CI −375, −311 pg/ml), whereas it increased (*p* = 0.04; +53 pg/ml; 95% CI +22, +84 pg/ml) with A6 (*p* < 0.001; Fig. 2e). HbA_1c_ decreased (*p* < 0.001) comparably with both regimens (−0.23%; 95% CI −0.27, −0.19% with A6 vs −0.25%; 95% CI −0.29, −0.20% with B2; *p* = 0.08; Fig. 2f).

### Whole-body insulin sensitivity

MCR increased (*p* < 0.001) comparably with both regimens (+0.45 ml kg^−1^ min^−1^; 95% CI +0.24, +0.67 ml kg^−1^ min^−1^ with A6 vs +0.52 ml kg^−1^ min^−1^; 95% CI +0.30, +0.74 ml kg^−1^ min^−1^ with B2; *p* = 0.8; Fig. 2g). OGIS increased in both regimens (*p* < 0.01), more with B2 (*p* = 0.01; +8.2 ml min^−1^ m^−2^; 95% CI +3.4, +13.1 ml min^−1^ m^−2^ with A6 vs +21 ml min^−1^ m^−2^; 95% CI +16.1, +26.0 ml min^−1^ m^−2^ with B2; Fig. 2h).

### Beta cell function

Insulin secretion at the reference level and glucose sensitivity increased (*p* < 0.05) comparably with both regimens. Rate sensitivity and potentiation factor also decreased (*p* < 0.05) comparably (Table 2).

### Plasma lipids

Triacylglycerols and LDL-cholesterol decreased comparably under both regimens. No significant change in total or HDL-cholesterol was observed in either regimen (Table 2).

### REE

REE decreased under both regimens (*p* < 0.001), with a trend toward a greater decrease with A6 (−453.1 kJ/day; 95% CI −524.3, −382.8 kJ/day with A6 vs −379.9 kJ/day; 95% CI −449.8, −310.9 kJ/day with B2; *p* = 0.3; Fig. 2i).

### Correlations

The decrease in HFC showed a strong positive correlation with the decrease in fasting plasma glucose (*r* = +0.56; *p* < 0.001). This association remained significant even after adjustment for changes in BMI (*r* = +0.28; *p* = 0.05).

Changes in glucose sensitivity and OGIS correlated negatively with changes in HFC (*r* = −0.28; *p* = 0.02 and *r* = −0.47; *p* < 0.001, respectively). After adjustment for changes in BMI, the correlations were no longer significant.

## Discussion

### Principal findings

This randomised crossover 24 week study examined the effect of frequency of meals on body weight, HFC, insulin resistance and beta cell function in type 2 diabetic patients. A comparison of the effect of six vs two meals (breakfast and lunch) with the same daily energy restriction (−2,092 kJ/day) and macronutrient content, each regimen lasting 12 weeks, demonstrated a superior effect of B2 on body weight, HFC, fasting plasma glucose, C-peptide, glucagon and OGIS. The effect of meal frequency on MCR and beta cell function was not significant.

### Findings in relation to other research

The superior effect of B2 on most of the variables studied supports our hypothesis and previous lines of evidence from animal models, observational studies and randomised trials [5, 7, 8, 12, 14].

Our results are in strong agreement with animal studies, which have demonstrated the glucose-lowering effects of intermittent fasting regimens (such as every other day fasting or fasting for 2 days a week). These regimens reduce blood glucose and insulin concentrations, improve glucose tolerance [4, 28], mainly due to increased insulin sensitivity [29], and extend the lifespan of laboratory animals [30].

Our data contradict the widely held opinion that eating more frequently is healthier than eating less frequent larger meals. Some studies have suggested that people who consume more snacks are less likely to be obese [31], but other large prospective studies have demonstrated that frequent snacking may lead to weight gain [32] and an increased risk of type 2 diabetes [33, 34] because of the higher energy intake, mainly from added sugars [35]. Furthermore, the reported benefits of more frequent meals are usually associated with meal frequencies exceeding those which might be translated into practical recommendations [36]. In fact, some of the early experiments with frequency of eating [37, 38] only underline the importance of fibre and low glycaemic index foods in the diet.

Studies involving individuals with type 2 diabetes are rather limited in both length and sample size. A longer term study, comparing the effect of three and nine meals daily (each period lasting 4 weeks), in 13 patients with type 2 diabetes, did not confirm the beneficial effects of increased meal frequency [39]. It has recently been demonstrated that, for glycaemic control, eating fewer larger meals rich in fibre instead of more smaller ones may be more beneficial for type 2 diabetic patients [10].

Ours is the first study into the effect of meal frequency on insulin resistance and HFC. MCR increased in response to both regimens, with a trend toward a greater increase with B2. The difference between the two regimens was not significant, probably due to a limited number of participants. OGIS increased in response to both regimens, more for B2. The increased insulin sensitivity positively influenced the decrease in fasting plasma glucose and HFC (although the HbA_1c_ level decreased comparably in both regimens) or, conversely, decreased HFC may have led to increased insulin sensitivity, because HFC is typically associated with insulin resistance (independent of BMI) [40], metabolic syndrome, type 2 diabetes and subclinical atherosclerosis [41]. In this context, the greater reduction in HFC in the B2 regimen is one of the most important results of our study.

We are also the first to observe an effect on fasting plasma concentrations of glucagon with a B2 regimen. Inappropriately elevated plasma glucagon concentrations play a role in dysregulated hepatic glucose production and abnormal glucose homeostasis in type 2 diabetes [42]. There is no treatment that specifically decreases glucagon levels. However, incretin-based agents reduce plasma glucagon, which contributes to their action to lower blood glucose [43]. In this regard, a decrease of glucagon with B2 in our study is a very positive finding.

### Possible mechanisms

The mechanisms responsible for a greater weight loss with the B2 regimen may be the trend toward a smaller decrease in REE with B2 that we measured (although the difference between regimens did not reach the threshold for statistical significance), together with a greater thermogenic response of larger meals, as documented by others [11].

Furthermore, some of the positive effects of intermittent fasting on glucose homeostasis may be mediated by the nervous system, mainly by the increased production of brain-derived neurotrophic factor, which increases the resistance of neurones to dysfunction and degeneration in animal models [3]. It is possible that overnight fasting between lunch and breakfast the next day elicited some of these effects.

Periods of fasting between meals may be even more important than the composition of the diet. In one experimental study, the timing of meals led to increased insulin sensitivity and decreased body weight in spite of the high fat content of the consumed diet [6]. The distribution of the meals is another important factor. Eating meals later in the day may also adversely influence the success of a weight loss therapy. This difference in weight loss success was not explained by differences in caloric intake, macronutrient distribution or energy expenditure [12]. A potential mechanism explaining this difference is that the timing of food intake can influence the circadian system [44]. The circadian system must continuously adapt to and synchronise our physiology with the environment [45]. A genetic variance in clock genes may be important in meal timing, possibly in part by changes in the recently demonstrated circadian control of hunger and appetite [46].

Another recent study demonstrated that a high carbohydrate and protein breakfast may prevent weight regain by reducing diet-induced compensatory changes in hunger, cravings and ghrelin suppression [47]. Although the mechanisms linking the timing of meals and the regulation of body weight are unknown, satiety hormones, such as leptin or ghrelin, may be involved [48]. Changes in the levels of these hormones by circadian misalignment could influence energy intake and expenditure [49].

### Weaknesses

The short duration of our study and provision of food precludes a generalisation of our study to free-living conditions. We provided all the meals during the whole study for one half of the participants in order to ensure the best possible compliance, yet we have to admit the possibility of a reduced energy intake with the B2 regimen, even though the energy intake reported by our participants was similar in both regimens. The dropout rates were also comparable for both regimens.

The sample of diabetic patients in our study was not representative: the duration of diabetes in our patients was quite short; all the participants were being treated by oral hypoglycaemic agents, with motivation not to initiate insulin therapy, and they were willing to make substantial changes in their lifestyle.

### Conclusions and research needs

In conclusion, a hypoenergetic diet (a restriction of 2,092 kJ/day) consumed as breakfast and lunch reduced body weight, HFC, fasting plasma glucose, C-peptide and glucagon, and increased calculated insulin sensitivity (OGIS), more than the same hypoenergetic diet (a similar restriction of 2,092 kJ/day) divided into six, more frequent, meals. MCR and beta cell function improved comparably in both regimens. These results suggest that eating two larger meals a day (breakfast and lunch) may be more beneficial for patients with type 2 diabetes than six smaller meals during the day. Novel therapeutic strategies should incorporate not only the energy and macronutrient content but also the frequency and timing of food. Further larger scale, long-term studies are essential before offering recommendations in terms of meal frequency.

## Abbreviations

A6: Six meals a day regimen
B2: Two meals a day regimen
HFC: Hepatic fat content
MCR: Metabolic clearance rate of glucose
OGIS: Oral glucose insulin sensitivity
REE: Resting energy expenditure

## References

[1] Visscher TL, Seidell JC (2001). The public health impact of obesity. Annu Rev Public Health.

[2] Mattson MP (2005). The need for controlled studies of the effects of meal frequency on health. Lancet.

[3] Mattson MP (2005). ENERGY intake, meal frequency, and health: a neurobiological perspective. Annu Rev Nutr.

[4] Anson RM, Guo Z, de Cabo R (2003). Intermittent fasting dissociates beneficial effects of dietary restriction on glucose metabolism and neuronal resistance to injury from calorie intake. Proc Natl Acad Sci U S A.

[5] Hatori M, Vollmers C, Zarrinpar A (2012). Time-restricted feeding without reducing caloric intake prevents metabolic diseases in mice fed a high-fat diet. Cell Metab.

[6] Sherman H, Genzer Y, Cohen R (2012). Timed high-fat diet resets circadian metabolism and prevents obesity. FASEB J.

[7] Howarth NC, Huang TT-K, Roberts SB (2007). Eating patterns and dietary composition in relation to BMI in younger and older adults. Int J Obes 2005.

[8] Duval K, Strychar I, Cyr M-J (2008). Physical activity is a confounding factor of the relation between eating frequency and body composition. Am J Clin Nutr.

[9] Bachman JL, Raynor HA (2012). Effects of manipulating eating frequency during a behavioral weight loss intervention: a pilot randomized controlled trial. Obesity (Silver Spring).

[10] Fernemark H, Jaredsson C, Bunjaku B (2013). A randomized cross-over trial of the postprandial effects of three different diets in patients with type 2 diabetes. PLoS One.

[11] Tai MM, Castillo P, Pi-Sunyer FX (1991). Meal size and frequency: effect on the thermic effect of food. Am J Clin Nutr.

[12] Garaulet M, Gómez-Abellán P, Alburquerque-Béjar JJ (2013). Timing of food intake predicts weight loss effectiveness. Int J Obes (Lond).

[13] Ruge T, Hodson L, Cheeseman J (2009). Fasted to fed trafficking of fatty acids in human adipose tissue reveals a novel regulatory step for enhanced fat storage. J Clin Endocrinol Metab.

[14] Purslow LR, Sandhu MS, Forouhi N (2008). Energy intake at breakfast and weight change: prospective study of 6,764 middle-aged men and women. Am J Epidemiol.

[15] Mann JI, de Leeuw I, Hermansen K (2004). Evidence-based nutritional approaches to the treatment and prevention of diabetes mellitus. Nutr Metab Cardiovasc Dis.

[16] Ferrannini E (1988). The theoretical bases of indirect calorimetry: a review. Metabolism.

[17] Hagströmer M, Oja P, Sjöström M (2006). The International Physical Activity Questionnaire (IPAQ): a study of concurrent and construct validity. Public Health Nutr.

[18] Baecke JA, Burema J, Frijters JE (1982). A short questionnaire for the measurement of habitual physical activity in epidemiological studies. Am J Clin Nutr.

[19] Pelikánová T, Smrcková I, Krízová J (1996). Effects of insulin and lipid emulsion on renal haemodynamics and renal sodium handling in IDDM patients. Diabetologia.

[20] Hájek M, Dezortová M, Wagnerová D (2011). MR spectroscopy as a tool for in vivo determination of steatosis in liver transplant recipients. Magma N Y N.

[21] Longo R, Pollesello P, Ricci C (1995). Proton MR spectroscopy in quantitative in vivo determination of fat content in human liver steatosis. J Magn Reson Imaging.

[22] van Cauter E, Mestrez F, Sturis J, Polonsky KS (1992). Estimation of insulin secretion rates from C-peptide levels. Comparison of individual and standard kinetic parameters for C-peptide clearance. Diabetes.

[23] Mari A, Tura A, Gastaldelli A, Ferrannini E (2002). Assessing insulin secretion by modeling in multiple-meal tests: role of potentiation. Diabetes.

[24] Mari A, Schmitz O, Gastaldelli A (2002). Meal and oral glucose tests for assessment of beta -cell function: modeling analysis in normal subjects. Am J Physiol Endocrinol Metab.

[25] Kahleova H, Mari A, Nofrate V (2012). Improvement in β-cell function after diet-induced weight loss is associated with decrease in pancreatic polypeptide in subjects with type 2 diabetes. J Diabetes Complicat.

[26] Meloun M, Hill M, Militký J, Kupka K (2000). Transformation in the PC-aided biochemical data analysis. Clin Chem Lab Med.

[27] Meloun M, Militký J, Hill M, Brereton RG (2002). Crucial problems in regression modelling and their solutions. Analyst.

[28] Pedersen CR, Hagemann I, Bock T, Buschard K (1999). Intermittent feeding and fasting reduces diabetes incidence in BB rats. Autoimmunity.

[29] Anson RM, Jones B, de Cabod R (2005). The diet restriction paradigm: a brief review of the effects of every-other-day feeding. Age (Dordr).

[30] Goodrick CL, Ingram DK, Reynolds MA (1982). Effects of intermittent feeding upon growth and life span in rats. Gerontology.

[31] Keast DR, Nicklas TA, O’Neil CE (2010). Snacking is associated with reduced risk of overweight and reduced abdominal obesity in adolescents: National Health and Nutrition Examination Survey (NHANES) 1999–2004. Am J Clin Nutr.

[32] van der Heijden AAWA, Hu FB, Rimm EB, van Dam RM (2007). A prospective study of breakfast consumption and weight gain among U.S. men. Obesity (Silver Spring).

[33] Mekary RA, Giovannucci E, Willett WC (2012). Eating patterns and type 2 diabetes risk in men: breakfast omission, eating frequency, and snacking. Am J Clin Nutr.

[34] Mekary RA, Giovannucci E, Cahill L (2013). Eating patterns and type 2 diabetes risk in older women: breakfast consumption and eating frequency. Am J Clin Nutr.

[35] Larson N, Story M (2013). A review of snacking patterns among children and adolescents: what are the implications of snacking for weight status?. Child Obes Print.

[36] Mann J (1997). Meal frequency and plasma lipids and lipoproteins. Br J Nutr.

[37] Jenkins DJ, Wolever TM, Vuksan V (1989). Nibbling versus gorging: metabolic advantages of increased meal frequency. N Engl J Med.

[38] Fabry P, Hejl Z, Fodor J (1964). The frequency of meals. Its relation to overweight, hypercholesterolaemia, and decreased glucose-tolerance. Lancet.

[39] Arnold L, Mann JI, Ball MJ (1997). Metabolic effects of alterations in meal frequency in type 2 diabetes. Diabetes Care.

[40] Seppälä-Lindroos A, Vehkavaara S, Häkkinen A-M (2002). Fat accumulation in the liver is associated with defects in insulin suppression of glucose production and serum free fatty acids independent of obesity in normal men. J Clin Endocrinol Metab.

[41] Juárez-Rojas JG, Medina-Urrutia AX, Jorge-Galarza E (2013). Fatty liver increases the association of metabolic syndrome with diabetes and atherosclerosis. Diabetes Care.

[42] D’Alessio D (2011). The role of dysregulated glucagon secretion in type 2 diabetes. Diabetes Obes Metab.

[43] Edwards KL, Stapleton M, Weis J, Irons BK (2012). An update in incretin-based therapy: a focus on glucagon-like peptide-1 receptor agonists. Diabetes Technol Ther.

[44] Garaulet M, Madrid JA (2010). Chronobiological aspects of nutrition, metabolic syndrome and obesity. Adv Drug Deliv Rev.

[45] Bienertová-Vasků J, Bienert P, Forejt M (2010). Genotype x nutrient association of common polymorphisms in obesity-related genes with food preferences and time structure of energy intake. Br J Nutr.

[46] Scheer FAJL, Morris CJ, Shea SA (2013). The internal circadian clock increases hunger and appetite in the evening independent of food intake and other behaviors. Obesity (Silver Spring).

[47] Jakubowicz D, Froy O, Wainstein J, Boaz M (2012). Meal timing and composition influence ghrelin levels, appetite scores and weight loss maintenance in overweight and obese adults. Steroids.

[48] Arble DM, Bass J, Laposky AD (2009). Circadian timing of food intake contributes to weight gain. Obesity (Silver Spring).

[49] Ma Y, Bertone ER, Stanek EJ (2003). Association between eating patterns and obesity in a free-living US adult population. Am J Epidemiol.

